# Capsular Contracture Rates in Subfascial and Subglandular Breast Augmentation With Smooth vs Textured Implants

**DOI:** 10.1093/asj/sjag017

**Published:** 2026-01-23

**Authors:** Erin N Abbott, Jordan Johnson, Nomongo Dorjsuren, Benjamin L Savitz, Emmanuel Giannas, Barite W Gutama, G Patrick Maxwell, Galen Perdikis, Louis L Strock, Allen Gabriel

## Abstract

Subfascial placement has been reported to reduce capsular contracture (CC) in primary breast augmentation. Given the shift toward smooth implants, it is unclear whether the perceived advantage reflects the surgical plane or historical implant selection. This study compares CC rates between subfascial and subglandular breast augmentation stratified by implant surface. A systematic review and meta-analysis were conducted. PubMed (National Institutes of Health, Bethesda, MD), Embase (Elsevier, Amsterdam, the Netherlands), Scopus (Elsevier), Cochrane Central Register of Controlled Trials (CENTRAL; Cochrane Library, London, UK), and Google Scholar (Alphabet, Inc., Mountain View, CA) were searched through April 2025. Studies reporting CC in primary augmentation with specified implant surface and plane were included. Random-effects models generated pooled incidences and odds ratios. Subgroup analyses evaluated studies published in the last decade with a mean follow-up duration of >24 months. Thirty-three studies met inclusion criteria. Across all years, studies comparing subfascial vs subglandular placement for smooth implants demonstrated no statistically significant difference in CC rates (7.2% vs 17.1%, *P* = .13). Studies evaluating textured implants similarly showed no significant difference between planes (0.9% vs 3.7%, *P* = .08). In studies published since 2015, smooth implants demonstrated comparable CC rates between subglandular and subfascial placement (8.2% vs 10.3%, *P* = .54). In studies with a mean follow-up of >24 months, no significant differences were observed for smooth implants, whereas textured implants demonstrated lower rates with subfascial placement. Microtextured implants demonstrated the lowest CC rates. When controlling for implant surface, CC rates do not differ significantly between subfascial and subglandular placement. Previously reported reductions in CC with subfascial augmentation appear driven by textured-implant cohorts. In contemporary smooth-implant practice, CC rates are similar across prepectoral planes.

**Level of Evidence:** 3 (Therapeutic) 
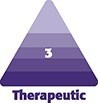

Breast augmentation is one of the most commonly performed aesthetic surgical procedures worldwide, with over 1.8 million procedures globally and ∼250,000 in the United States in 2023.^1,2^ Prepectoral implant placement, first refined in breast reconstruction, has also gained traction in aesthetic augmentation as advances in implant design and soft-tissue support have improved safety and reduced overall complication rates, including capsular contracture (CC). The subfascial plane, located between the pectoralis major muscle and its overlying fascia, has gained popularity for its proposed benefits of reduced CC rates, fewer animation deformities, and improved aesthetic contour.^3,4^

Although the current literature generally supports subfascial augmentation, most studies reported textured implants or did not distinguish between implant surface types. Textured implants were preferred in the prepectoral space because of their association with lower CC rates.^5-7^ In the United States, however, surgical practice has shifted almost entirely to smooth implants following US Food and Drug Administration (FDA) safety concerns regarding breast implant–associated anaplastic large cell lymphoma (BIA-ALCL).^8,9^

Given this shift, it is important to reevaluate whether the reduced CC rates reported with subfascial placement are attributable to the plane itself or to the implant surface characteristics that predominated in earlier studies. To address this question, a systematic review and meta-analysis was conducted to compare CC rates between smooth and textured implants placed in the subfascial and subglandular planes. It was hypothesized that smooth implants would demonstrate comparable CC rates between planes in the prepectoral space and that the perceived advantage of subfascial placement is more closely related to implant surface rather than pocket selection, particularly with the latest generation of smooth implants.

## METHODS

This systematic review and meta-analysis was conducted in accordance with the Preferred Reporting Items for Systematic Reviews and Meta-Analyses (PRISMA) guidelines.^10^ The protocol was registered with the International Prospective Register of Systematic Reviews (PROSPERO; CRD420251007019). Preliminary searches were performed by a single author (E.N.A.) to refine and validate the final search strategy. PubMed (National Institutes of Health, Bethesda, MD), Embase (Elsevier, Amsterdam, the Netherlands), Scopus (Elsevier), Cochrane Central Register of Controlled Trials (CENTRAL; Cochrane Library, London, United Kingdom), and Google Scholar (Alphabet, Inc., Mountain View, CA) were systematically searched on April 7, 2025 (Supplemental Table 1). Screening was performed by 2 authors (E.N.A. and N.D.). Disagreements were resolved by a third author (B.L.S.), with escalation to senior authorship if required (A.G.). Records were managed and screened using Covidence systematic review software (Veritas Health Innovation, Melbourne, Australia).

The date range for the study was from January 1990 to April 2024. Studies reporting the incidence of CC following primary breast augmentation were eligible for inclusion. Reports limited to breast reconstruction, gender-affirming surgery, or revision cases were excluded. Case reports, narrative or systematic reviews, and nonhuman studies were also excluded. Only studies specifying both implant surface type and plane of placement were considered.

Extracted data included first author, year of publication, country of the first author's affiliation, study design, sample size, implant type and manufacturer, incision type, plane of placement, length of follow-up, and incidence of CC as defined by Baker grade III or IV on physical examination.

CC rates were pooled by implant surface and type, and a random-effects meta-analysis was performed, with 95% CIs. Subgroup analyses compared studies from the last 10 years with the entire cohort, given advancements in smooth-implant manufacturing over the past decade. Studies published within the last 10 years were included to ensure relevance to contemporary implant technology. Additional subgroup analyses were performed, restricting inclusion to studies with a mean follow-up duration >24 months to account for the time-dependent development of CC. Subgroup analyses based on fewer than 3 studies per comparison were not interpreted as confirmatory, given the instability of variance estimates and limited power to detect true between-group differences. Odds ratio analyses were performed where comparative data were available. Data and statistical analysis were performed using R (v4.4.1; R Foundation for Statistical Computing, Vienna, Austria). Study quality was assessed using the Risk of Bias in Nonrandomized Studies of Interventions (ROBINS-I).^11^

## RESULTS

**Figure 1.**
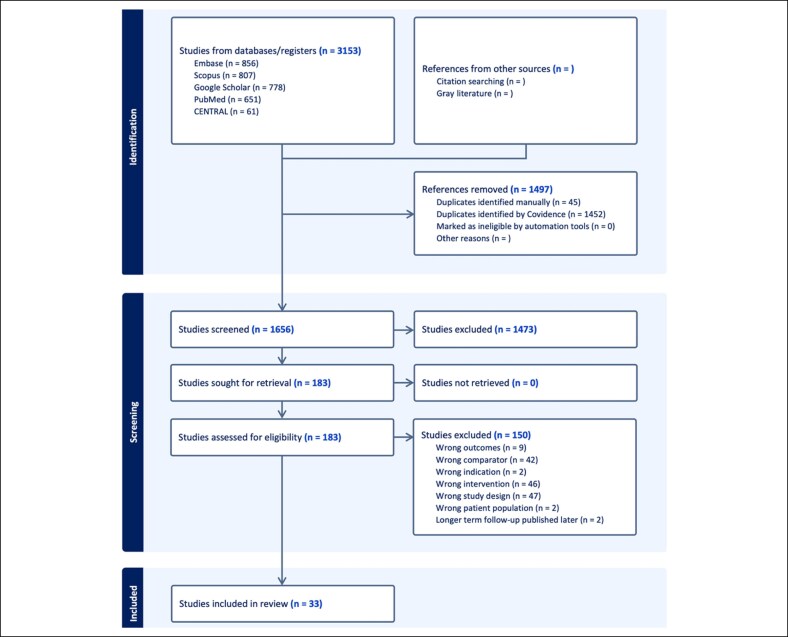
PRISMA flow diagram showing identification, screening, eligibility assessment, and inclusion of studies in the systematic review.

A total of 3153 studies were identified through database searches: Embase (*n* = 856), Scopus (*n* = 807), Google Scholar (*n* = 778), PubMed (*n* = 651), and CENTRAL (*n* = 61). After removal of 1497 duplicates, 1656 studies remained for screening. Of these, 1473 were excluded based on title and abstract review. The full texts of 183 studies were assessed; 150 were excluded for reasons including wrong intervention (*n* = 46), wrong study design (*n* = 47), insufficient subgroup data (*n* = 42), and other predefined exclusion criteria. A total of 33 studies met the inclusion criteria and were included in the final analysis (Figure 1).^5,12-43^

Characteristics of the studies included are presented in Supplemental Table 2. The date of publication ranged from 1990 to 2025, with 17 published since 2015. Studies originated from 15 countries across 5 continents; the United States was most represented with 11 publications (33%). Included studies comprised 2 randomized controlled trials (RCTs), 5 prospective multicenter, 6 prospective single-center, 11 retrospective, and 9 technique papers reporting outcomes. Sample sizes for primary augmentation cohorts ranged from 7 to 5059 patients. The mean patient age was 33.2 years (range, 18-66 years). Follow-up duration ranged from 4 days to 10 years. CC rates by implant plane and surface type are summarized in Supplemental Table 3.

**Figure 2.**
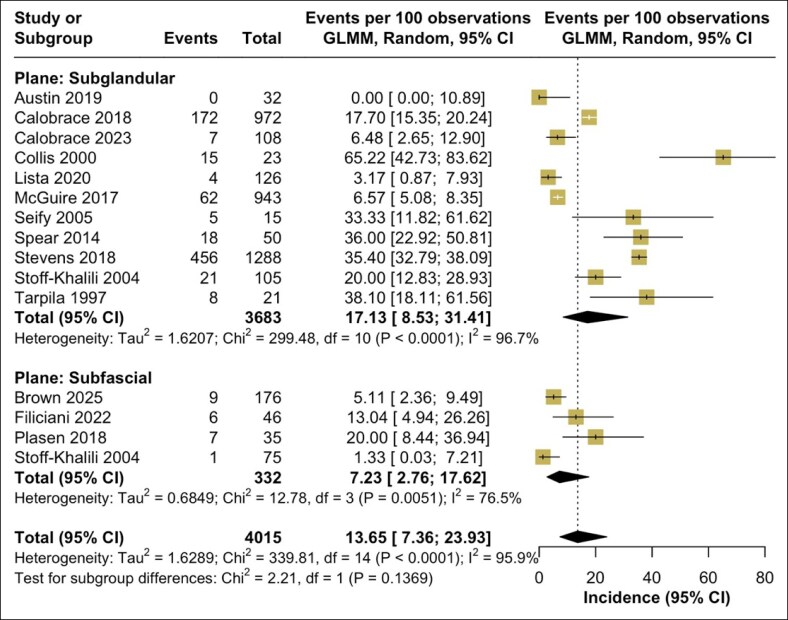
Pooled capsular contracture rates for smooth implants in subglandular, subfascial, and combined prepectoral placement across all included studies. Forest plot demonstrating the pooled incidence of capsular contracture among smooth implants by plane of placement.

**Figure 3.**
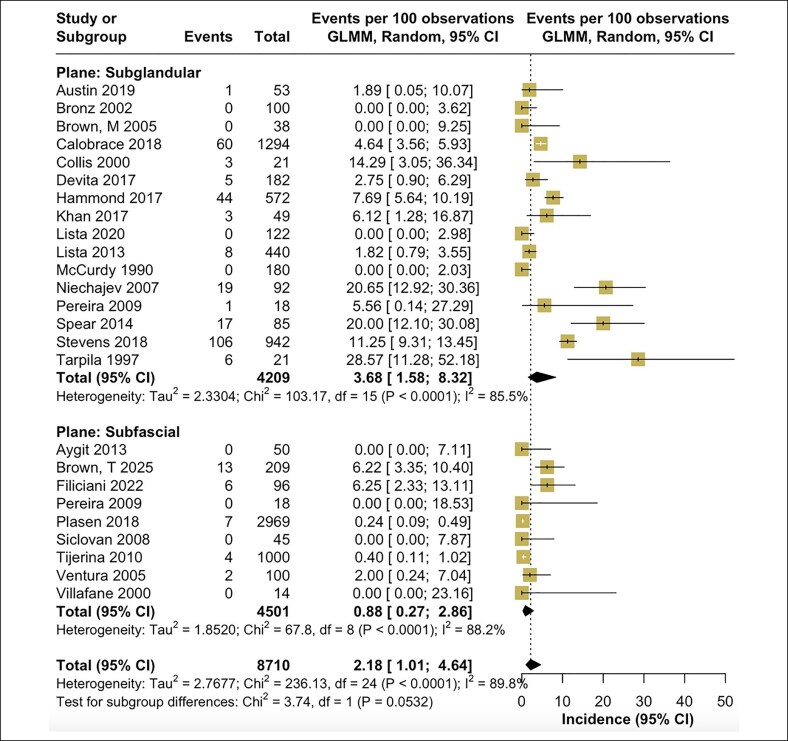
Pooled capsular contracture rates for textured implants in subglandular, subfascial, and combined prepectoral placement across all included studies. Forest plot demonstrating the pooled incidence of capsular contracture among textured implants by plane of placement.

A meta-analysis was subsequently performed. Among studies evaluating smooth implants, the pooled incidence of CC was 17.1% (95% CI, 8.5%-31.4%) for subglandular and 7.2% (95% CI, 2.8%-17.6%) for subfascial placement. Although the subfascial plane demonstrated a lower numerical rate, the difference was not statistically significant (*P* = .14). Heterogeneity was high across both subgroups (*I*^2^ = 96.7% and 76.5%, respectively; Figure 2). Among studies evaluating textured implants, the pooled CC incidence was 3.7% (95% CI, 1.6%-8.3%) for subglandular and 0.9% (95% CI, 0.3%-2.9%) for subfascial placement. The difference approached but did not reach statistical significance (*P* = .053; Figure 3).

**Figure 4.**
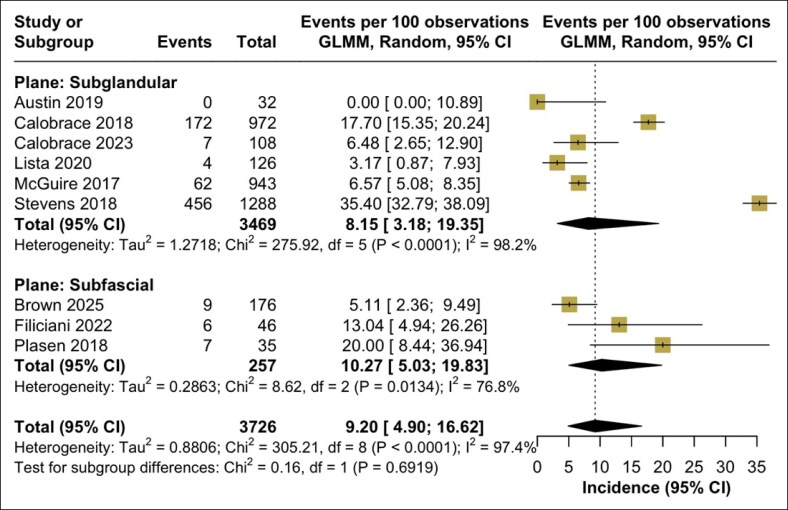
Pooled capsular contracture rates for smooth implants in subglandular, subfascial, and combined prepectoral placement among studies published since 2015. Forest plot reflecting contemporary smooth-implant outcomes by plane.

**Figure 5.**
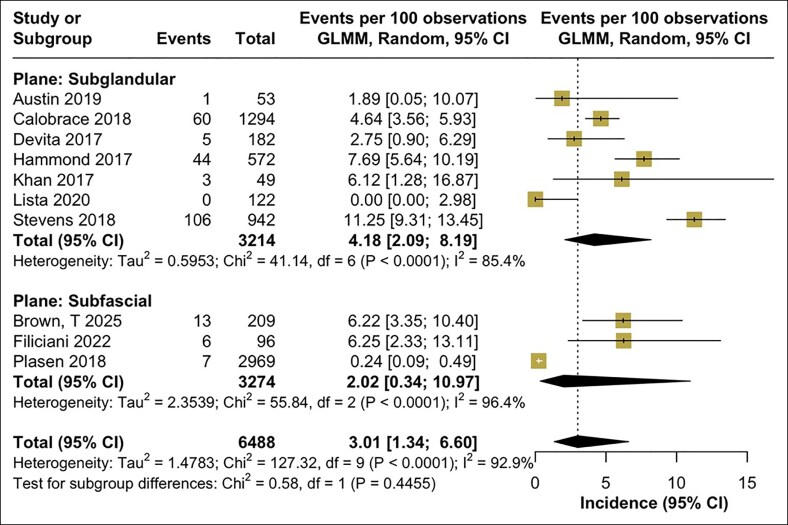
Pooled capsular contracture rates for textured implants in subglandular, subfascial, and combined prepectoral placement among studies published since 2015. Forest plot reflecting contemporary textured-implant outcomes by plane.

**Figure 6.**
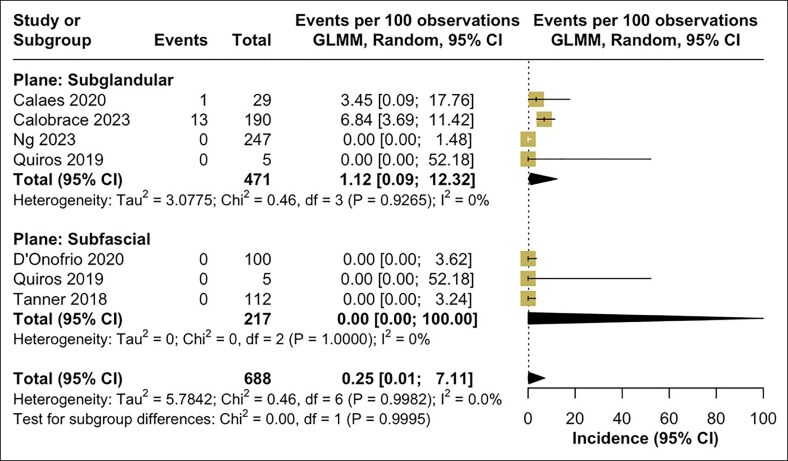
Pooled capsular contracture rates for microtextured implants in subglandular, subfascial, and combined prepectoral placement among studies published since 2015. Forest plot demonstrating capsular contracture incidence for microtextured devices by plane.

In studies published since 2015, pooled incidence of CC for smooth implants was 8.2% (95% CI, 3.2%-19.4%) in the subglandular plane and 10.3% (95% CI, 5.0%-19.8%) in the subfascial plane, with no significant difference between groups (*P* = .69; Figure 4). Pooled CC incidence for textured implants was 4.2% (95% CI, 2.1-8.9) in the subglandular plane and 2.0% (95% CI, 0.3-11.0) in the subfascial plane, with no significant subgroup difference (*P* = .45; Figure 5). Lastly, pooled CC incidence for microtextured implants was 1.1% (95% CI, 0.1-12.3) in the subglandular plane and 0.0% (95% CI, 0.0-100.0) in the subfascial plane, with no significant difference between groups (*P* = .99; Figure 6).

Overall, the CC rate for implants placed in the prepectoral space was 13.7% for smooth and 2.2% for textured. During the last decade, CC rates within the prepectoral space were 9.2% for smooth, 3.01% for textured, and 0.25% for microtextured implants. There were no statistically significant differences in pooled CC rates between pre-2015 and post-2015 studies for either surface type (smooth, *P* = .07; textured, *P* = .40).

**Figure 7.**
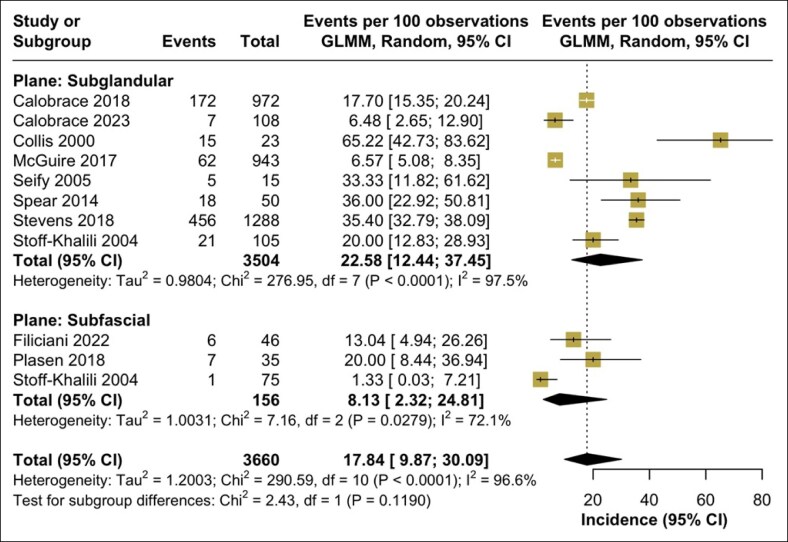
Pooled capsular contracture rates for smooth implants in subglandular, subfascial, and combined prepectoral placement among studies with mean follow-up >24 months. Forest plot demonstrating capsular contracture incidence for smooth implants by plane across all included study years.

**Figure 8.**
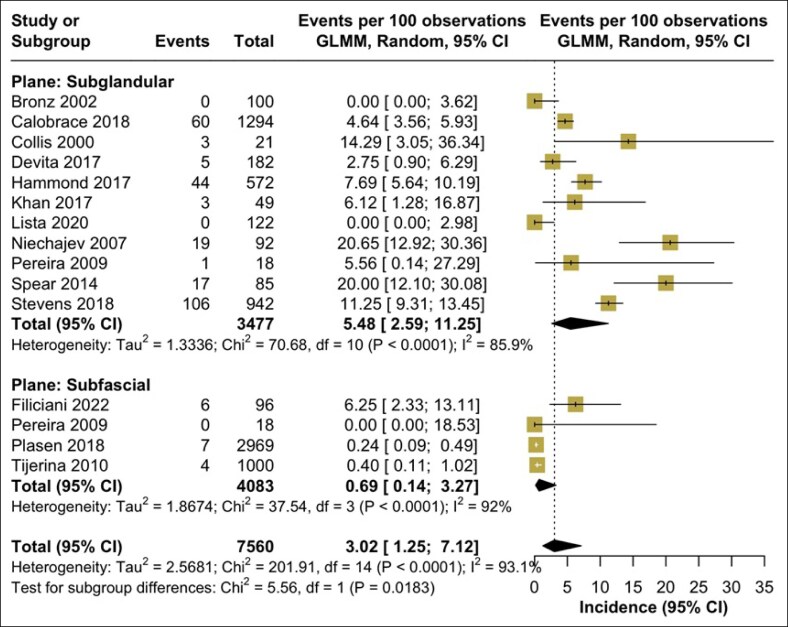
Pooled capsular contracture rates for textured implants in subglandular, subfascial, and combined prepectoral placement among studies with mean follow-up >24 months. Forest plot demonstrating capsular contracture incidence for textured implants by plane across all included study years.

When analyses were restricted to studies with a mean follow-up >24 months, pooled CC incidence among smooth implants was 22.6% (95% CI, 12.4%-37.5%) in the subglandular plane and 8.1% (95% CI, 2.3%-24.8%) in the subfascial plane. The difference between planes was not statistically significant (*P* = .12; Figure 7). Among textured implants with >24 months of mean follow-up, pooled CC incidence was 5.5% (95% CI, 2.6%-11.3%) in the subglandular plane and 0.7% (95% CI, 0.1%-3.3%) in the subfascial plane. In this subgroup, subfascial placement was associated with a significantly lower incidence of CC compared with subglandular placement (*P* = .018; Figure 8).

**Figure 9.**
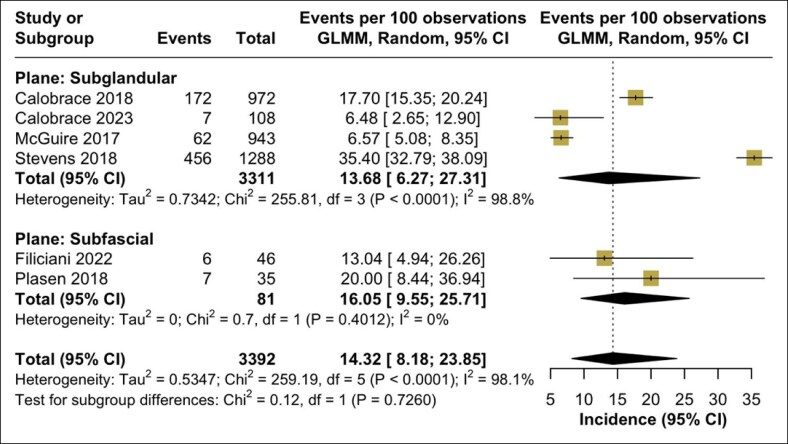
Pooled capsular contracture rates for smooth implants in subglandular, subfascial, and combined prepectoral placement among studies published since 2015 with mean follow-up >24 months. Forest plot demonstrating capsular contracture incidence for smooth implants by plane in contemporary studies with extended follow-up.

**Figure 10.**
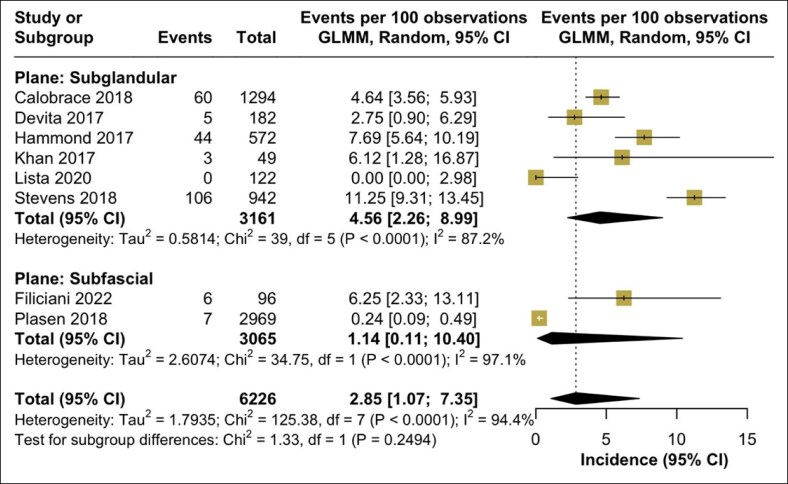
Pooled capsular contracture rates for textured implants in subglandular, subfascial, and combined prepectoral placement among studies published since 2015 with mean follow-up >24 months. Forest plot demonstrating capsular contracture incidence for textured implants by plane in contemporary studies with extended follow-up.

In studies published since 2015 with a mean follow-up of at least 24 months, CC incidence among smooth implants was 13.7% (95% CI, 6.3%-27.3%) in the subglandular plane and 16.1% (95% CI, 9.6%-25.7%) in the subfascial plane, with no statistically significant difference between planes (*P* = .73; Figure 9). For textured implants, CC was 4.6% (95% CI, 2.3%-9.0%) in the subglandular plane and 1.1% (95% CI, 0.1%-10.4%), which was not significantly different (*P* = .25; Figure 10).

Among the 33 included studies, one was assessed as having an overall low risk of bias; the remainder were judged to have moderate (*n* = 21) or serious risk (*n* = 11; Supplemental Figure 1, available online at https://doi.org/10.1093/asj/sjag017). The most common sources of bias were confounding and missing data, with over half of the studies rated as moderate or serious risk in these domains. Bias related to the selection of participants and classification of interventions was generally moderate, reflecting limitations in study design and incomplete reporting of baseline comparability. Bias because of deviations from intended interventions and measurement of outcomes was less frequent and typically rated as low risk, reflecting consistent use of Baker grading or comparable clinical assessments across studies. Only a minority of studies explicitly addressed selective reporting, contributing to uncertainty in overall risk assessment. Although many studies demonstrated low or moderate risk in individual domains, the overall risk of bias was rated serious in most cases (Supplemental Figure 1).

## DISCUSSION

In this systematic review and meta-analysis, the idea that subfascial placement confers a lower risk of CC for all implants was not supported. Across all studies, CC rates varied primarily by implant surface rather than by plane. Earlier studies showed large numerical differences between planes, but these did not reach statistical significance and were largely driven by textured-implant cohorts. Smooth implants demonstrated a pooled CC incidence of ∼10% across all prepectoral placements, with no significant difference between subfascial and subglandular planes. In contrast, textured implants consistently exhibited lower CC rates, whereas microtextured implants showed the lowest rates overall, although data for the latter was limited. In analyses limited to the past decade, CC incidence declined across all implant types, although these reductions were not statistically significant, suggesting improvements in implant manufacturing and surgical technique rather than protective effects of fascial elevation.

When analyses were restricted to studies with longer follow-up durations, textured implants demonstrated a significantly lower incidence of CC with subfascial placement compared with subglandular placement in the overall cohort. However, this association was not observed in contemporary studies with extended follow-up, and smooth implants continued to demonstrate similar CC rates between subfascial and subglandular planes, including in studies published within the past decade.

Across all studies included, the consistent CC rate within implant type underscores the presumption that plane selection has a significant impact on CC rates. This supports our argument that the decreased rate in CC is likely a result of advancements in implant technology and not an advantage of the subfascial plane. The overall quality of evidence was low, with most studies rated at moderate or serious risk of bias, reflecting the predominance of retrospective designs and incomplete control for confounding.

A subgroup analysis was completed for studies published in the last decade. Modern fifth-generation implants with modifications in gel cohesivity, shell thickness, and surface texture were introduced during this period, making earlier devices less representative of current clinical use.^44-47^ Within the past 10 years, there has been a shift toward highly cohesive, form stable silicone gel implants, when compared with previous decades.^44,48,49^ This timeframe also captures the sharp decline in the use of textured implants following their association with BIA-ALCL, making smooth implants the implant of choice for many surgeons.^45,48-50^

### Follow-Up Duration and Interpretation of Capsular Contracture Risk

Because CC is a delayed complication whose incidence increases over time, inclusion of studies with shorter mean follow-up durations represents a fundamental limitation of the existing CC literature. When studies with longer mean follow-up were examined, CC rates remained similar between subfascial and subglandular placement for smooth implants, reinforcing that plane selection alone does not meaningfully alter contracture risk in contemporary practice. Although textured implants demonstrated a lower CC rate with subfascial placement in the overall long-term subgroup, this finding was based on a limited number of studies and was not reproduced in contemporary cohorts, underscoring the difficulty of drawing durable conclusions from underpowered long-term analyses.

A closer examination of individual studies further illustrates the impact of follow-up duration on reported CC rates. Although some studies provide stratified or extended follow-up data, others report very short minimum follow-up intervals or heterogeneous follow-up between implant cohorts. Calaes et al and Brown attempted to report longitudinal outcomes or cohort-level follow-up distributions, allowing more informed interpretation of CC risk over time.^16,17^ In contrast, other studies such as Lista et al and Ng et al (2023) report minimum follow-up durations of only weeks to months or unequal follow-up between smooth and textured implant groups.^25,26,29^ Earlier studies may also reflect historical limitations in the understanding of CC timing, as well as older implant designs and surgical paradigms. Collectively, these issues underscore the need for standardized minimum follow-up thresholds of at least 24 to 36 months, exclusion of inadequately followed patients when CC is a primary outcome, and stratified reporting of contracture incidence over time to support meaningful long-term comparisons.

### Historical Context and Evolution of Subfascial Plane

The subfascial plane for breast augmentation was popularized by Graf in 2003 as a new pocket intended to create a more natural breast contour by avoiding implant distortion from pectoralis major contraction and providing additional anterior soft-tissue camouflage compared with subglandular placement.^51^ Proponents suggest that it combines elements of both submuscular and subglandular placement, offering reduced implant animation compared with submuscular augmentation.^31^ It has been theorized that positioning the upper pole of the implant between the muscle and fascia provides additional stabilization and may help prevent displacement over time. The plane is also described as allowing for greater soft-tissue coverage and a more natural upper-pole contour, particularly in thin patients.^35,42,52^ Advocates further propose that subfascial placement may avoid injury to the superficial fascia, including the Cooper ligament system and lymphatic structures; preservation of Cooper ligaments could theoretically help maintain native breast position and reduce ptosis, while sparing lymphatic structures might decrease postoperative edema.^42^ Some small series have also reported less immediate postoperative pain and faster recovery compared with subglandular or submuscular placement, although these findings have not been consistently reproduced.^41^

Despite these proposed advantages, the subfascial plane is not without limitations. Anatomical studies confirm the presence of the pectoral fascia, but it is thin (0.2-1.14 mm) and highly variable in integrity.^42,53^ The fascia is particularly inconsistent inferiorly, making the layer difficult to reliably identify and maintain during dissection.^54,55^ Because the pocket must be created within this variable and delicate layer, asymmetry or contour irregularities may occur if the plane is inconsistently developed or incompletely dissected. In patients with limited upper-pole soft-tissue coverage at baseline, implant edge palpability or visible rippling may still occur whether the implant is placed above the pectoralis or in a subfascial position, resulting in suboptimal aesthetic outcomes.^16^ Thus, although the plane offers theoretical advantages, its independent contribution to long-term results remains uncertain. Overall, although the subfascial technique gained international popularity, its use in the United States remains limited.^56^

### Evidence From Pro-Subfascial Literature

Literature has suggested that the subfascial plane is associated with lower rates of CC compared with alternative planes. However, this finding appears largely attributable to the use of textured implants rather than the plane itself. Because many studies do not specify the implant type used, it is often unclear whether differences in CC reflect implant surface characteristics or the subfascial technique.

Kerfant et al conducted a retrospective review of 156 patients who underwent subfascial implant placement with fat grafting using textured implants and reported CC rates by Baker grade (BII: 2.6%, BIII: 2%).^57^ Aygit et al performed a retrospective case series of 27 patients who underwent transaxillary subfascial augmentation with microtextured implants and reported CC rates of 16% (Baker grade II) and 0% (Baker grade III/IV) at 21 months of follow-up.^13^ Similarly, Brown reviewed 783 patients who underwent subfascial augmentation with round textured implants and reported a CC rate of 6.48%, lower than that reported for subglandular placement.^16^

Graf et al first introduced the subfascial plane as a new pocket for breast implant placement, applying the technique in 263 augmentations over 3 years. They reported favorable aesthetic outcomes with low CC rates (Baker grade II, 2.3%) and improved soft-tissue coverage compared with subglandular placement. However, all implants used were BIOCELL textured (Allergan, Dublin, Ireland), follow-up was limited, and the study lacked a comparison group.^51^ In a later double-blinded, intra-patient randomized trial, Graf et al compared subfascial and subglandular placement in 20 patients and reported a lower CC rate at 5 years (0% vs 16%), although all implants were textured and the study was limited by its small sample size.^58^

Gould et al published a systematic review and meta-analysis of 26 studies (3743 patients), most of which used textured implants, reporting an overall CC rate of 1.01% and a statistically significant reduction in CC in the subfascial group.^4^ However, the inclusion of observational studies introduced selection bias related to surgeon preference, and implant-type comparisons were not performed. Yuan et al conducted a comprehensive review comparing subfascial and subglandular planes and reported significantly lower rates of CC, hematoma, and rippling with subfascial placement (all *P* < .05). Nonetheless, the authors’ narrow search strategy, inconsistencies in the PRISMA diagram, and high risk of bias across included studies limit the reliability of their conclusions. Several included studies excluded patients who developed complications, and the reported effect sizes were small, suggesting limited clinical relevance.^3^

Taken together, these studies have contributed to the perception that subfascial placement reduces the risk of CC. However, this association is largely confounded by implant surface and methodological bias. When controlling implant type and follow-up duration, there is little evidence to support that the subfascial plane independently decreases CC risk.

### Capsular Contracture in Smooth vs Textured Breast Implants in the Subglandular Plane

Subglandular implant placement provides a versatile approach to breast augmentation, allowing the procedure to be performed without disrupting the pectoralis major muscle. This avoids the animation deformity commonly seen with subpectoral or dual-plane augmentation and preserves the natural dynamics of the chest wall. The use of smooth implants in the subglandular plane has long been scrutinized because of reports of higher CC rates compared with textured implants.

In a systematic review of 6 RCTs comparing textured and smooth implants for subglandular augmentation (235 patients, 470 breasts), textured implants were associated with lower rates of CC by Baker grade at 1 year (relative risk [RR], 4.16; 95% CI, 1.58-10.96), 3 years (RR, 7.25; 95% CI, 2.42-21.69), and 7 years (RR, 2.98; 95% CI, 0.86-10.37).^7^ Similarly, Barnsley et al conducted a meta-analysis of 7 RCTs evaluating textured vs smooth implants and found a significantly lower risk of CC in the subglandular plane with textured devices.^59^ A subsequent RCT by Namnoum et al confirmed a reduced risk of CC with textured implants regardless of subpectoral or subglandular placement.^60^ In a number-needed-to-treat analysis, 7 to 9 patients would require a textured implant to prevent 1 Baker grade III and IV contracture over 10 years.

However, many of the primary studies included in these analyses did not demonstrate statistically significant associations between implant texture and CC reduction. For instance, 4 of the 7 RCTs analyzed in Barnsley et al failed to reach statistical significance. Moreover, implant devices have advanced considerably since these earlier studies, which may limit the applicability of their findings to current practice.

More recent studies comparing smooth and textured implants in subglandular placement have yielded conflicting results. Allergan's Natrelle Core Study, a prospective cohort of 715 patients enrolled between 1999 and 2000 with 10-year follow-up, found no statistically significant difference in CC rates between smooth (37.0%) and textured (20.2%) subglandular implants (*P* = .058).^36^ Conversely, Sientra's Core Study, another prospective cohort of 1788 patients enrolled beginning in 2002, demonstrated a significantly lower CC rate with textured implants (11.3%) compared with smooth (35.4%) at 10 years (*P* = .0007).^37^ Notably, these cohorts preceded the widespread adoption of “minimal-touch” techniques (insertion sleeves, nipple shields, bloodless dissection, and optimized incision lengths), now associated with decreased capsule formation.

Lista et al examined 526 patients who underwent primary subglandular augmentation between 2010 and 2017 using either smooth (*n* = 212) or textured (*n* = 314) round silicone gel implants. Among these, 248 underwent augmentation alone, and 278 underwent augmentation mastopexy. At an average follow-up of 756 days for textured and 461 days for smooth implants, CC occurred in 5 textured cases (1.59%) and 7 smooth cases (3.30%), a difference that was not statistically significant (*P* = .20).^25^ However, the relatively short follow-up period may have underestimated the true long-term incidence of contracture.

Collectively, textured implants have historically shown lower CC rates than smooth implants in the subglandular plane. Yet, this difference diminishes when modern implant designs and contemporary surgical techniques are considered. Further investigation is warranted to clarify the role of implant surface in subglandular placement, particularly in the context of evolving technology and current smooth-only clinical practice.

### Capsular Contracture in Smooth vs Textured Breast Implants in the Subfascial Plane

The available literature on CC rates in smooth vs textured implants placed in the subfascial plane remains conflicting and methodologically limited. In a retrospective review of 253 patients (506 implants) from January 2017 to July 2019, Filiciani et al found that smooth-surface implants placed in the subfascial plane demonstrated a 4-fold higher risk of CC than textured implants within the same plane (odds ratio, 4.4; 95% CI, 1.6-12.4).^22^ Although it may be expected for textured implants to have a higher CC rate because of increased susceptibility to bacterial contamination and biofilm formation, this contrasts with what is observed in clinical practice.^61^ This is perhaps another example of how modern techniques, such as minimal-touch principles, and modern devices play a more significant role in CC development than plane selection.

Derby and Codner reviewed data from the 3 major US manufacturers’ core studies evaluating long-term complication profiles up to 10 years following primary breast augmentation. Across all 3 manufacturers (Mentor [Santa Barbara, CA], Allergan, and Sientra [Irvine, CA]), textured implants exhibited lower overall CC rates compared with smooth implants. However, considerable variability existed among implant types: Mentor smooth and Siltex round implants demonstrated a 12.1% CC rate at 12 years of follow-up, whereas Allergan smooth and Biocell round implants exhibited a 19.1% rate at 10 years.^45^ Notably, CC rates increased over time, emphasizing the importance of long-term follow-up in assessing implant outcomes.

More recently, Bistoni et al retrospectively reviewed 72 patients (46 primary and 26 secondary cases) who underwent subfascial augmentation mastopexy using smooth round implants supported by a P4HB scaffold. With a mean follow-up of 24.8 months, the study reported no cases of recurrent ptosis, bottoming out, implant displacement, or CC.^62^ However, the exclusion of patients with follow-up <1 year or significant weight variation (±8 kg) limits the generalizability of these findings. Similarly, D’Onofrio evaluated smooth implants in subfascial augmentation with a crossed fascial sling and reported no cases of CC, although the majority of patients were followed for only 6 months.^20^ Ng et al also reported no instances of CC in a retrospective review of subfascial augmentation using a new smooth, round, opaque implant (PERLE, GC Aesthetics; Dublin, Ireland), but the mean follow-up was limited to 18 months.^29^

Overall, findings are variable and often constrained by small samples, short follow-up, and inconsistent CC definitions. Although some studies report no cases of contracture, others indicate higher rates than those seen with textured implants. The evidence base remains limited by small sample sizes, short follow-up periods, and inconsistent definitions of CC. The present meta-analysis quantifies these differences and indicates the apparent advantage of subfascial placement is largely dependent on implant surface rather than pocket selection alone.

### Capsular Contracture With Microtextured Implants in the Subfascial and Subglandular Planes

Microtextured, or nanotextured, implants are designed to combine the advantages of both smooth and textured devices—offering potentially lower rates of CC without the reported risk of BIA-ALCL associated with macrotextured surfaces. Studies have suggested that CC rates with microtextured implants are comparable with or lower than those observed with either smooth or textured implants.^63^

Tanner conducted a retrospective case series of 214 patients who underwent augmentation with round microtextured implants manufactured in France. A standardized surgical protocol was used, including inframammary incisions and perioperative antibiotics. At 20 months of follow-up, CC occurred in 0% of primary augmentations and 2.3% of revisions, for an overall rate of 0.9%.^39^ The low CC incidence was attributed to both optimized surgical technique and the use of microtextured implants.

Aygit et al reported a series of 27 patients who underwent microtextured implant–based subfascial augmentation through a transaxillary approach. The authors modified the traditional subfascial technique to achieve complete subfascial coverage rather than partial superficial dissection, reporting a 16% incidence of Baker grade II CC over an average of 21 months.^13^ Caplin conducted an open-label clinical trial evaluating long-term outcomes of round vs shaped microtextured implants. In primary augmentation, Baker grade III/IV CC rates were 11.3% for round and 3.4% for shaped implants (*P* < .0001). In revision augmentation, rates were 24.4% and 15.6%, respectively (*P* < .05). The reduced CC rates were attributed to the shaped implant surface and a standardized educational protocol for participating surgeons.^64^

Overall, microtextured implants demonstrate promising outcomes in reducing CC rates for primary augmentation. However, generalizability remains limited by the small number of studies and variations in implant manufacturing. Additionally, the longest reported follow-up period for microtextured implants is ∼21 months, which may miss late-onset CC.^65,66^

### Capsular Contracture in Smooth vs Textured Breast Implants in Submuscular Plane

Submuscular breast implant placement has consistently been reported as a protective factor against CC.^67^ A previous systematic review found no significant difference in CC rates between smooth and textured implants when placed submuscularly (3%-14% vs 6%-20%, respectively).^68^ Despite these advantages, trends in aesthetic breast augmentation have increasingly shifted toward prepectoral placement to minimize animation deformity, postoperative pain, and disruption of the pectoralis major muscle. Accordingly, this review focused on prepectoral approaches where CC risk remains higher, and implant surface may be more clinically relevant.

### Clinical Implications

The perceived lower rate of CC associated with subfascial implant placement, as reported in the literature, appears to be largely attributable to the historical use of textured implants. Following the 2019 US FDA recall of macrotextured implants because of the associated risk of BIA-ALCL, this benefit is no longer applicable to current US practice.^69^ When focusing on smooth implants, the predominant implant type used in the US data, data indicate that CC rates in the subfascial plane are equivalent to those in the subglandular plane.^50^ Ultimately, pocket selection should be individualized but cannot be made independently of implant surface characteristics.

### Limitations

Several methodological strengths support the validity of these findings: a broad search across multiple databases, a larger screened cohort than previous reviews, and stratification by both implant surface and plane. Nevertheless, important limitations include the predominance of retrospective designs, incomplete control for confounding, and significant heterogeneity that limits interpretation and generalizability. Many of the reports included were technique papers, which are prone to publication and positive bias, potentially overestimating favorable outcomes. Additionally, although the Baker classification system was reported regularly, this grading remains subjective and susceptible to interobserver variability.

Additionally, several included studies reported a relatively short follow-up period of 6 to 12 months. CC is a time-dependent complication that may not manifest or progress to clinically significant Baker grade III/IV contracture within the first 12 months after implantation. Previous literature suggests that ∼66% of CC cases present within the first 12 months after surgery, whereas up to 79% occur within the first 24 months.^70^ As a result, studies with limited follow-up likely underestimate the true long-term incidence of CC. Despite the addition of subgroup analyses using a minimum mean follow-up time of 24 months, relatively few studies report mean follow-up beyond 24 months, and even fewer extend beyond 3 years, limiting the ability to draw definitive conclusions regarding long-term CC risk.

Lastly, surgical incision represents a potential confounding variable that could not be adequately controlled for in this analysis. Although incision type is a known risk factor for CC, most included studies only reported the distribution of incision approaches but did not stratify rates of contracture by incision type.^71,72^ As a result, it was not possible to evaluate the effect of incision type on CC outcomes. In addition, heterogeneity and limited reporting of perioperative practice variables, including implant handling protocols, antimicrobial use, drain placement, and no-touch techniques, precluded meaningful subanalysis. Despite these limitations, this review offers valuable evidence-based insight to guide surgical decision making regarding implant placement and surface selection.

### Future Directions

Prospective studies evaluating smooth implants in the subfascial plane with standardized procedures, consistent outcome assessment, and US-relevant data are needed. Large, multi-institutional databases would mitigate bias and improve external validity. Comparing historical textured-implant cohorts with contemporary smooth-implant data could quantify the impact of the 2019 FDA recall on CC incidence. Finally, standardizing CC assessment beyond the subjective Baker scale, potentially incorporating objective clinical or imaging-based criteria, and adjusting for adjuncts such as soft-tissue support and fat grafting will clarify their roles in modulating CC.

## CONCLUSIONS

This systematic review and meta-analysis found no significant difference in CC rates between subfascial and subglandular implant placement when stratified by implant surface. The numerical differences reported in earlier literature appear to reflect the predominant use of textured devices rather than an independent benefit of fascial elevation, particularly when included studies were limited to the last decade and with longer-term follow-up. In contemporary practice, where smooth implants are standard, CC rates in the prepectoral space are similar regardless of whether the fascia is elevated. Improvements in implant design, surgical technique, and minimal-touch principles are likely to contribute more to reductions in contracture than the plane selection in the prepectoral space.

## Supplemental Material

This article contains supplemental material located online at https://doi.org/10.1093/asj/sjag017.

## Supplementary Material

sjag017_Supplementary_Data
