# Supplementary material for: Capsular Contracture Rates in Subfascial and Subglandular Breast Augmentation With Smooth vs Textured Implants
Source: Aesthet Surg J. 2026 Jan 23;46(9):979–92. doi: 10.1093/asj/sjag017 (PMC13481099; doi:10.1093/asj/sjag017)
Supplement: Supplementary file 1 [file sjag017_supplementary_data.zip › Supplemental Table 2.docx]

Supplemental Table 2

| **Author (Year)** | **Country** | **Study Design** | **N (Patients)** | **N (Breasts)** | **Age (Range, years)** | **Mean Follow-up (Range, months)** | **Incision type** | **Implant type** | **Manufacturer** |
| --- | --- | --- | --- | --- | --- | --- | --- | --- | --- |
| Austin,  2019 | Canada | Technique with outcomes | 85 |  | 34  (17-62) | 13.4  (0.4-86.7) | Periareolar | TX | NR |
| Aygit,  2013 | Turkey | Technique with outcomes | 27 | 50 | 27.3  (19-32) | 21  (7-28) | Transaxillary | TX | Mentor |
| Bronz,  2002 | Switzerland | Prospective, single center | 50 | 100 | 40.9  (23-60) | NR  (48 - 108) | Inframammary (62)  Periareolar (38) | TX | McGhan |
| Brown, M,  2005 | Canada | Retrospective | 118 | 235 | NR | 21  (16-36) | Inframammary(95)  Periareolar(22) | TX | Inamed Aesthetics |
| Brown, T,  2020 | Australia | Retrospective | 385 |  | SM: 34.9 (18-62)  TX: 33.7 (18-57) | SM: 13.7 (6.2 - 22.7)TX: 15.5 (6.5 -23.3) | NR | SM and TX | Nagor Ltd |
| Calaes,  2020 | Brazil | Randomized clinical trial | 58 |  | MCTX: 23.9 (19-50) | 12 (100%), 24 (66%) 36 (28%) | Inframammary | MCTX | Lifesil |
| Calobrace,  2018 | USA | Prospective, single center | 2565 |  | 36  (18-66) | 120 | Periareolar (29%) Inframmamary (71%) | SM and TX | Sientra |
| Calobrace,  2023 | USA | Prospective, multicenter | 4046 |  | 32  (22-73) | 72 | Periareolar (899), inframammary (5826),  Mastopexy incision (1030), Transaxillary (334),  not provided (1) | TX and MCTX | Sientra |
| Collis,  2000 | England | RCT | 40 |  | 30  (21-44) | 120 | Inframammary | TX | Mentor |
| D'Onofrio,  2020 | UAE | Technique with outcomes | 100 |  | NR  (23-35) | 6 | Periareolar | MCTX | Motiva |
| DeVita,  2017 | Italy | Technique with outcomes | 182 |  | 43.5  (28-59) | 24 | Periareolar with inverted T mastopexy | TX | Mentor and Johnson & Johnson |
| Filiciani,  2022 | Argentina | Retrospective | 253 | 506 | 33.5  (18-58) | 24 | Inframammary | TX and SM | Mentor, Nagor, Allergan, Polytech,  and Eurosilicon |
| Hammond,  2017 | USA | Prospective, multicenter | 572 |  | 35.8 | 120 | NR | TX | Sil TX, Mentor |
| Khan,  2017 | England | Retrospective | 117 |  | 32.2  (18-53) | 79  (18 -144) | Inframammary | TX | NR |
| Lista,  2013 | USA | Retrospective | 440 | 880 | 35  (17-62) | 13  (0.3 to 120) | Inframammary | TX | Allergan |
| Lista,  2020 | USA | Retrospective | 526 |  | 36  (17.3-62.9) | TX: 24  SM: 15 | Inframammary | SM and TX | Allergan and Mentor |
| McCurdy,  1990 | USA | Retrospective |  | 585 | NR | 6 to 12 | Mostly Periareolar | SM andTX | SM: NR,TX: Meme, Biocel, Sil TX |
| McGuire,  2017 | USA | Prospective, multicenter | 5059 | 10091 | 36  (18-82) | 49 | Inframammary (91%) | TX | Allergan |
| Ng,  2023 | England | Retrospective | 290 |  | 32  (19-62) | 18  (6-30) | Inframammary (80%) | MCTX | GC Aesthetics |
| Niechajev,  2007 | Sweden | Prospective, single center | 80 | 134 | 28  (17-51) | 60  (48-72) | Inframammary (69), Periareolar (10), Transaxillary (1) | TX | McGhan and Eurosilicone Ver TX |
| Pereira,  2009 | Brazil | Technique with outcomes | 53 |  | 24  (17-32) | 36 | Transaxillary | TX | NR |
| Plasen,  2018 | Thailand | Prospective, single center | 3004 |  | 29  (19-61) | 120 | Transaxillary Endoscopic | TX and SM | Mentor, Allergan |
| Quiros,  2019 | Costa Rica | Prospective, single center | 35 |  | 31.5  (21-51) | 72 | Inframammary (97%) | SM | Motiva |
| Seify,  2005 | USA | Retrospective | 44 |  | 32  (19-57) | 34  (28-40) | Inframammary and Areolar | SM | Mentor |
| Siclovan,  2008 | Saudi Arabia | Technique with outcomes | 45 |  | NR | NR | Inframammary | TX | Mentor |
| Spear,  2014 | USA | Prospective, multicenter | 455 |  | 34 | 120 | Inframammary and Areolar | SM and TX | Natrelle and Biocell |
| Stevens,  2018 | USA | Prospective, multicenter | 1116 | 2230 | 36 | 120 | Inframammary and Areolar | TX | Sientra |
| Stoff-Khalili, 2004 | USA | Retrospective | 328 |  | 34  (17-48) | 35 (subfascial)  43 (subglandular) | Periareolar | TX and SM | McGhan and CUI/McGhan |
| Tanner,  2018 | England | Retrospective | 126 |  | 40 | 20 | Inframammary | TX | Groupe SEBBIN SAS  Boissy l’Aillerie |
| Tarpila,  1997 | Sweden | Prospective, single center | 21 | 42 | 33  (22-48) | 12 | Inframammary | TX and SM | Allegran and McGhan |
| Tijerina,  2010 | Mexico | Technique with outcomes | 1000 |  | NR | 120 | Inframammary | TX | NR |
| Ventura,  2005 | Argentina | Technique with outcomes | 100 |  | NR | NR | Inferior Periareolar | TX | McGhan |
| Villafane,  2000 | England | Technique with outcomes | 7 | 14 | NR | 6 | Endoscopic Transaxillary | TX | NR |
