# Supplementary material for: Capsular Contracture Rates in Subfascial and Subglandular Breast Augmentation With Smooth vs Textured Implants
Source: Aesthet Surg J. 2026 Jan 23;46(9):979–92. doi: 10.1093/asj/sjag017 (PMC13481099; doi:10.1093/asj/sjag017)
Supplement: Supplementary file 1 [file sjag017_supplementary_data.zip › Supplemental Table 3.docx]

Supplemental Table 3. Capsular contracture rates by implant plane and surface type

| **Author,**  **Year** | **Following by breast or by patient:** | **Number of SM subglandular** | **Number of SM subglandular with CC** | **Number of TX**  **subglandular** | **Number of TX subglandular with CC** | **Number of MCTX subglandular** | **Number of MCTX subglandular with CC** | **Number of SM subfascial** | **Number of SM subfascial with CC** | **Number of TX subfascial** | **Number of TX subfascial with CC** | **Number of MCTX subfascial** | **Number of MCTX subfascial with CC** |
| --- | --- | --- | --- | --- | --- | --- | --- | --- | --- | --- | --- | --- | --- |
| Austin,  2019 | Patient | 32 | 0 | 53 | 1 |  |  |  |  |  |  |  |  |
| Aygit,  2013 | Breast |  |  |  |  |  |  |  |  | 50 | 0 |  |  |
| Bronz,  2002 | Breast |  |  | 100 | 0 |  |  |  |  |  |  |  |  |
| Brown, M, 2005 | Patient |  |  | 38 | 0 |  |  |  |  |  |  |  |  |
| Brown, T, 2020 | Patient |  |  |  |  |  |  | 176 | 9 | 209 | 13 |  |  |
| Calaes,  2020 | Patient |  |  |  |  | 29 | 1 |  |  |  |  |  |  |
| Calobrace, 2023 | Patient | 108 | 7 |  |  | 190 | 13 |  |  |  |  |  |  |
| Calobrace, 2018 | Breast | 972 | 172 | 1294 | 60 |  |  |  |  |  |  |  |  |
| Collis,  2000 | Patient | 23 | 15 | 21 | 3 |  |  |  |  |  |  |  |  |
| DeVita,  2017 | Patient |  |  | 182 | 5 |  |  |  |  |  |  |  |  |
| D'Onofrio, 2020 | Patient |  |  |  |  |  |  |  |  |  |  | 100 | 0 |
| Filiciani, 2022 | Breast |  |  |  |  |  |  | 46 | 6 | 168 | 6 |  |  |
| Hammond, 2017 | Patient |  |  | 572 | 44 |  |  |  |  |  |  |  |  |
| Khan,  2017 | Patient |  |  | 49 | 3 |  |  |  |  |  |  |  |  |
| Lista,  2020 | Patient | 126 | 4 | 122 | 0 |  |  |  |  |  |  |  |  |
| Lista,  2013 | Patient |  |  | 440 | 8 |  |  |  |  |  |  |  |  |
| McCurdy, 1990 | breast |  |  | 180 | 0 |  |  |  |  |  |  |  |  |
| McGuire, 2017 | Patient | 943 | 62 |  |  |  |  |  |  |  |  |  |  |
| Ng,  2023 | Patient |  |  |  |  | 247 | 0 |  |  |  |  |  |  |
| Niechajev, 2007 | Breast |  |  | 92 | 19 |  |  |  |  |  |  |  |  |
| Pereira,  2009 | Patient |  |  | 18 | 1 |  |  |  |  | 18 | 0 |  |  |
| Plasen,  2018 | Patient |  |  |  |  |  |  | 35 | 7 | 2969 | 7 |  |  |
| Quiros,  2019 | Patient |  |  |  |  | 5 | 0 |  |  |  |  | 5 | 0 |
| Seify,  2005 | Patient | 15 | 5 |  |  |  |  |  |  |  |  |  |  |
| Siclovan, 2008 | Patient |  |  |  |  |  |  |  |  | 45 | 0 |  |  |
| Spear,  2014 | Patient | 50 | 18 | 85 | 17 |  |  |  |  |  |  |  |  |
| Stevens, 2018 | Patient | 1288 | 456 | 942 | 106 |  |  |  |  |  |  |  |  |
| Stoff-Khalili, 2004 | Patient | 105 | 21 |  |  |  |  | 75 | 1 |  |  |  |  |
| Tanner,  2018 | Patient |  |  |  |  |  |  |  |  |  |  | 112 | 0 |
| Tarpila,  1997 | Patient | 8 | 21 | 6 | 21 |  |  |  |  |  |  |  |  |
| Tijerina, 2010 | Patient |  |  |  |  |  |  |  |  | 1000 | 4 |  |  |
| Ventura, 2005 | Patient |  |  |  |  |  |  |  |  | 100 | 2 |  |  |
| Villafane, 2000 | Patient |  |  |  |  |  |  |  |  | 14 | 0 |  |  |
