# Supplementary material for: Capsular Contracture Rates in Subfascial and Subglandular Breast Augmentation With Smooth vs Textured Implants
Source: Aesthet Surg J. 2026 Jan 23;46(9):979–92. doi: 10.1093/asj/sjag017 (PMC13481099; doi:10.1093/asj/sjag017)
Supplement: Supplementary file 1 [file sjag017_supplementary_data.zip › SuppTable1.docx]

Supplemental Table 1. Search Terms

| Database | Search Terms |
| --- | --- |
| Embase (Ovid SP) | (breast reconstruction/ or breast implant/ or ("breast augmentation" or "breast implants" or "breast reconstruction" or (breast* adj3 (implant* or augment* or reconstruct*)) or (subfascial or plane)).mp.) and (textur* or smooth).mp. and ("capsular contracture" or Baker* or complication*).mp. [mp=title, abstract, heading word, drug trade name, original title, device manufacturer, drug manufacturer, device trade name, keyword heading word, floating subheading word, candidate term word] |
| Scopus | ALL=(((“breast augmentation” OR “breast implants” OR “breast reconstruction” OR (breast* AND (implant* OR augment* OR reconstruct*)) OR subfascial OR plane) AND (textur* OR smooth) AND (“capsular contracture” OR Baker* OR complication*))) |
| PubMed | (breast implant[MeSH Terms]) OR (((“breast augmentation” OR “breast implants”) OR (breast* AND (implant* OR augment* )) OR (subfascial OR subglandular OR prepectoral OR retrofascial OR subaponeurotic)) AND (textur* OR smooth) AND (“capsular contracture” OR Baker* OR complication*) |
| Google Scholar | ("breast augmentation" OR "breast implants" OR "breast reconstruction") AND("subfascial" AND ("textured" OR "texture" OR "smooth") AND ("capsular contracture" OR "Baker") |
| Cochrane | ("breast augmentation" OR "breast implants" OR "breast reconstruction" OR (breast* NEAR/3 (implant* OR augment* OR reconstruct*)) OR subfascial OR plane) AND (textur* OR smooth) AND ("capsular contracture" OR Baker* OR complication*) |
